# Blood eosinophils levels in a Colombian cohort of biomass-and tobacco-related COPD patients

**DOI:** 10.3389/fmed.2024.1321371

**Published:** 2024-05-13

**Authors:** Olga Milena García Morales, Alejandra Cañas-Arboleda, María Nelcy Rodríguez Malagón, Javier Leonardo Galindo Pedraza, Paola Rodríguez Torres, Violeta Rosa Avendaño Morales, Andrés Leonardo González-Rangel, Carlos A. Celis-Preciado

**Affiliations:** ^1^Service of Pneumology, Department of Internal Medicine, Hospital Universitario Fundación Santa Fe de Bogotá, Bogotá, Colombia; ^2^Service of Pneumology, Department of Internal Medicine, Hospital Universitario San Ignacio, Bogotá, Colombia; ^3^Faculty of Medicine, Pontificia Universidad Javeriana, Bogotá, Colombia; ^4^Department of Epidemiology and Biostatistics, Pontificia Universidad Javeriana, Bogotá, Colombia; ^5^Department of Respiratory Medicine, Hospital Universitario Mayor - Méderi, Bogotá, Colombia; ^6^Javesalud IPS, Bogotá, Colombia; ^7^Medical Affairs, GlaxoSmithKline, Bogotá, Colombia

**Keywords:** chronic obstructive pulmonary disease, smoking, biomass, eosinophils, phenotype, Colombia

## Abstract

**Introduction:**

Chronic obstructive pulmonary disease (COPD) is a major cause of illness and death among adults. In 2019, the Global Initiative for Chronic Obstructive Lung Disease (GOLD) strategy incorporated blood eosinophils as a biomarker to identify patients at increased risk of exacerbations which, with the history of exacerbations during the previous year, allows identification of patients who would benefit from anti-inflammatory treatment to reduce the risk of future exacerbations. The aim of this study was to describe demographic and clinical characteristics, eosinophil counts, and exacerbations in a cohort of COPD patients stratified by clinical phenotypes (non-exacerbator, frequent exacerbator, asthma-COPD overlap) in a Colombian cohort at 2600 meters above sea level.

**Methods:**

A descriptive analysis of a historical cohort of patients with a confirmed diagnosis of moderate to severe COPD (FEV_1_/FVC < 0.7 and at least one risk factor for COPD) from two specialized centers with comprehensive disease management programs was performed from January 2015 to March 2019. Data were extracted from medical records 1 year before and after the index date.

**Results:**

200 patients were included (GOLD B: 156, GOLD E: 44; 2023 GOLD classification); mean age was 77.9 (SD 7.9) years; 48% were women, and 52% had biomass exposure as a COPD risk factor. The mean FEV_1_/FVC was 53.4% (SD 9.8), with an FEV_1_ of 52.7% (20.7). No differences were observed between clinical phenotypes in terms of airflow limitation. The geometric mean of absolute blood eosinophils was 197.58 (SD 2.09) cells/μL (range 0 to 3,020). Mean blood eosinophil count was higher in patients with smoking history and frequent exacerbators. At least one moderate and one severe exacerbation occurred in the previous year in 44 and 8% of patients, respectively; during the follow-up year 152 exacerbations were registered, 122 (80%) moderate and 30 (20%) severe. The highest rate of exacerbations in the follow-up year occurred in the subgroup of patients with the frequent exacerbator phenotype and eosinophils ≥300 cells/μL.

**Discussion:**

In this cohort, the frequency of biomass exposure as a risk factor is considerable. High blood eosinophil count was related to smoking, and to the frequent exacerbator phenotype.

## 1 Introduction

Chronic obstructive pulmonary disease (COPD) is a complex and heterogeneous lung condition, with different phenotypic clinical presentations, which causes a high burden of morbidity and mortality in adults (1–3).

The overall prevalence of COPD ranges from approximately 6 to 12% worldwide (4–6). Male sex (odds ratio [OR] 2.1 [95% CI 1.8–2.3]), smoking (current OR 3.2 [95% CI 2.5–4.0] or ever smoker OR 2.3 [95% CI 2.0–2.5]), body mass index (BMI) of less than 18.5 kg/m^2^ (OR 2.2 [95% CI 1.7–2.7]), biomass exposure (OR 1.4 [95% CI 1.2–1.7]), and occupational exposure to dust or smoke (OR 1.4 [95% CI 1.3–1.6]) are risk factors for COPD (4). COPD in non-smokers constitutes almost one-third of all cases, and a substantial proportion of those are related to outdoor, occupational, and household air pollution, especially from biomass cookstove use in low-and middle-income countries (7).

In Colombia, the PREPOCOL (Prevalence of COPD in five Colombian cities) study reported a prevalence of 8.9% in the general population of adults aged 40 years and older (8). In this study, older age, male gender, history of tuberculosis, smoking, very low education level, and wood smoke exposure were factors related to COPD, something of interest for countries with similar economic conditions (8).

In recent years, the assessment of COPD patients has focused on using multidimensional indexes, phenotypes, and treatable traits to identify patients at increased risk of having poor health outcomes, such as acute exacerbations (9). An exacerbation of COPD is defined as an acute worsening of respiratory symptoms that is often associated with increased local and systemic inflammation and may lead to disease progression, higher rates of rehospitalization, increased healthcare costs, poorer quality of life, and mortality (10, 11). Type 2 inflammation, characterized by the involvement of eosinophils, is known to play a significant role in exacerbations and disease progression in COPD. As such, quantifying eosinophil levels in the blood has emerged as a potential predictor for future exacerbations in patients with stable COPD (12). Blood eosinophil count has proved to be a biomarker that may help identify responsiveness to inhaled corticosteroids (ICS); however, its effectiveness as a predictive biomarker of future exacerbations is weak in comparison with the history of previous exacerbations (13). Patients with frequent exacerbations require special attention to prevent complications arising from each acute event (14, 15).

COPD phenotypes represent distinct clinical and biological characteristics, contributing to an enhanced comprehension of the condition’s inherent heterogeneity and offering opportunities for personalized therapeutic approaches. Some common phenotypes include: non-exacerbator, frequent exacerbator (patients experiencing frequent acute exacerbations, often associated with increased inflammation and accelerated disease progression), chronic bronchitis (productive cough more than 3 months per year in two or more consecutive years), emphysematous (emphysema confirmed on imaging), and Asthma-COPD Overlap (ACO) (patients show characteristics of both asthma and COPD, such as variable airflow limitation and bronchial hyperresponsiveness) (16). The recognition of these phenotypes may help in tailoring of treatment strategies and interventions for better management of COPD.

Longitudinal COPD cohorts in low-and middle-income countries are scarce, leading to a gap in knowledge about the frequency of exacerbations and the feasibility of treatable trait-based strategies in most real-life settings worldwide. We aimed to describe demographic and clinical characteristics (including eosinophil counts), exacerbation frequency and hospitalizations during 1 year of follow-up in a cohort of COPD patients in Colombia, stratified by clinical phenotypes (non-exacerbator, frequent exacerbator, and ACO).

## 2 Materials and methods

### 2.1 Study design and participants

Descriptive study of a historical cohort of COPD patients from two centers with comprehensive disease management programs in Bogotá, Colombia, between January 1, 2015, and March 31, 2019.

Patients aged 40 years or older with a confirmed diagnosis of COPD, defined as the presence of respiratory symptoms (dyspnea, cough or wheezing), spirometry with obstructive pattern (forced expiratory volume in the first second to forced vital capacity ratio [FEV_1_/FVC] < 0.7), and the identification of at least one COPD risk factor (smoking, biomass or occupational exposure to dust or fumes) were included. At least one baseline blood eosinophil count, measured outside an exacerbation event, had to be available in each case for their inclusion.

We excluded patients with a history of other chronic lung diseases (silicosis, interstitial lung disease or pulmonary tuberculosis), pulmonary eosinophilia (acute or chronic eosinophilic pneumonia, hyper-eosinophilic syndrome, eosinophilic granulomatosis with polyangiitis or allergic bronchopulmonary aspergillosis), concurrent pulmonary neoplasm or incomplete medical records during the follow-up period (except COPD-related death).

### 2.2 Data collection and definitions

Demographic data (age, sex), baseline clinical characteristics (weight, height, BMI, modified Medical Research Council [mMRC] dyspnea classification, post-bronchodilator FEV_1_, post-bronchodilator FVC, FEV_1_/FVC, reversibility, comorbidities, prescribed treatment, and blood eosinophil levels), risk factors (smoking status, biomass and occupational exposure) recorded exacerbations (during 1 year after and 1 year before the index date), and inhaled therapy (at 6 and 12 months after the index date) were extracted from medical records and systematically collected using REDCap version 11.1.1 (Vanderbilt, Nashville, TN, USA).

Given the changes that the GOLD classification had during and after the study years, the most recent 2023 GOLD ABE classification was used for all study patients based on the original information present in the clinical records (17).

Elements of previous classifications such as categories of airflow limitation were also used in the analysis.

Exacerbations were defined as any worsening of respiratory symptoms requiring changes in maintenance medication, including antimicrobial therapy, oral corticosteroids, or inhaled therapy. Moderate exacerbations were defined as an unscheduled outpatient or emergency department visit, not requiring hospitalization, treated with antimicrobial therapy or oral corticosteroids for at least 3 days. Severe exacerbations were defined as those requiring hospitalization.

Based on exacerbation history and comorbidities, patients were classified into the following clinical phenotypes: non-exacerbator (one or fewer moderate exacerbations in the previous year and no severe exacerbations), frequent exacerbator (two or more moderate exacerbations or one severe in the previous year) and asthma-COPD overlap (ACO), defined as previous or current diagnosis of asthma (subtype 1) or evidence of reversibility (≥ 300 mL in FVC or FEV_1_) on spirometry (subtype 2).

The eosinophil threshold values to be considered as potential predictors of exacerbations were extracted from the literature, focusing on data derived from extensive multicenter cohorts presented by Yu et al. These cohorts utilized longitudinal data from two independent studies, namely, the Genetic Epidemiology of COPD (COPDGene) that included 1,553 patients, and the Evaluation of COPD Longitudinally to Identify Predictive Surrogate Endpoints (ECLIPSE) study, which had 1,895 participants. Both studies revealed an elevated risk of exacerbations associated with an eosinophil count of 300 cells/μL or higher, particularly among subjects with a history of frequent exacerbations. In multivariable logistic regression models, the eosinophil cutoff of 300 cells/μL demonstrated the highest sensitivity (72.6%) and specificity (66.0%) in identifying the risk of having one or more self-reported exacerbation (18, 19). Conversely, the Global Initiative for Chronic Obstructive Lung Disease (GOLD) has considered blood eosinophil counts <100 cells/μL in COPD patients as a predictor of poor inhaled corticosteroid (ICS) responsiveness (17, 20).

### 2.3 Statistical analysis

Patients who met the inclusion criteria were selected. Analyses were performed on a population rather than a probability sample; no statistical tests were performed for population-based inference of the results. Population size was estimated after a feasibility analysis at the participant institutions.

Descriptive analysis of variables of interest was performed to report absolute and relative frequencies of demographic, clinical, and laboratory data (including eosinophil levels), and exacerbation events. Continuous and discrete variables were expressed by the corresponding measures of central tendency (means or medians, as appropriate) and variability (standard deviations or interquartile ranges). To assess whether the dataset follows a normal distribution, the Shapiro–Wilk statistical test was used. For blood eosinophils counts, a log-normal distribution was used for absolute values and a Gaussian distribution for relative values.

A *post hoc* regression analysis was performed to measure the association between eosinophil levels and exacerbation rate, adjusted by clinically important covariates identified from the literature. A Poisson model was carried out using the number of total (moderate and severe) exacerbations in the observation year as outcome, and a high blood eosinophil count (≥ 300 cells/μL) as the exposition of interest. The potential confounders tested were frequent exacerbator phenotype, sex, smoking status (past or current exposition) and all interactions between these covariates and high eosinophils. A complete model was first assembled and then simplified using variation of the high eosinophil regression coefficient and clinical interpretability as permanence criteria. Only statistically significant interactions (*p*-value <0.05) were kept. The model assumptions (frequency of non-events, mean and variance of the outcome’s distribution) and the model dispersion were evaluated. All statistical analyses were performed using Stata version 14.0 (StataCorp LP, TX, USA).

### 2.4 Ethics

The study was approved by the Ethics and Research Committee of each center (Hospital Universitario San Ignacio and Javesalud IPS), in accordance with local and global regulations on research with human subjects. The study investigators guaranteed the anonymity and confidentiality of the information extracted from the patients’ medical records.

## 3 Results

### 3.1 Patient characteristics

This cohort consisted of 200 patients diagnosed with COPD; their baseline clinical characteristics are summarized in Table 1. Most patients were elderly (88% of patients were 70 years of age or older). The median Charlson Comorbidity Index was 6 (IQR 3); the most common comorbidity was arterial hypertension (79%), followed by obesity (34%) and diabetes (23%). Asthma was recorded in 10% of the patients.

**Table 1:** Baseline characteristics of the study population.

Sex, female (*n*, %)	96	48.0
Age, years (mean, SD)	77.9	7.4
Body mass index		
Normal weight (18.5 to 24.9 Kg/m^2^)	64	32.0
Overweight (25 to 29.9 Kg/m^2^)	69	34.5
Obesity (≥30 Kg/m^2^)	67	33.5
GOLD 2017 COPD classification		
GOLD B	156	78.0
GOLD C	3	1.5
GOLD D	41	20.5
GOLD 2023 COPD classification		
GOLD B	156	78.0
GOLD E	44	22.0
mMRC dyspnea scale		
1	3	1.5
2	145	72.5
3	52	26.0
GOLD airflow limitation severity		
GOLD 1	22	11.0
GOLD 2	78	39.0
GOLD 3	74	37.0
GOLD 4	26	13.0
BODEx score		
0–2 points	87	43.5
3–4 points	86	43.0
5 points or more	27	13.5
Charlson Comorbidity Index (median, IQR)	6.0	3.0
Comorbidities		
Arterial hypertension	158	79.0
Diabetes mellitus	46	23.0
Chronic kidney disease	41	20.5
Congestive heart failure	36	18.0
Cancer	22	11.0
Asthma	20	10.0
Sleep apnea	19	9.5
Cerebrovascular disease	19	9.5
Myocardial infarction	17	8.5
COPD risk factors		
Smoking only	57	28.5
Biomass only	48	24.0
Occupational only	12	6.0
Biomass and smoking	28	14.0
Occupational and smoking	26	13.0
Biomass and occupational	14	7.0
Biomass, occupational and smoking	15	7.5
COPD clinical phenotype		
Non-exacerbator	156	78.0
Frequent exacerbator	44	22.0
Asthma-COPD Overlap	27	13.5
Post-bronchodilator lung function		
FEV_1_, L (mean, SD)	1.0	0.3
FEV_1_, % predicted (mean, SD)	52.7	20.7
FVC, L (mean, SD)	1.9	0.6
FVC, % predicted (mean, SD)	73.4	22.9
FEV_1_/FVC, % predicted (mean, SD)	53.4	9.8
Reversibility, mL (median, IQR)	100.0	90.0
Reversibility, % (median, IQR)	9.0	17.0
Positive reversibility	31	15.5

IQR, interquartile range; SD, standard deviation; GOLD, Global Initiative for Chronic Obstructive Lung Disease; mMRC, modified Medical Research Council; BODEx, Body mass index; airflow Obstruction, Dyspnea, Exacerbations.

### 3.2 Blood eosinophil levels

The baseline absolute geometric mean of blood eosinophil count was 197.58 cells/μL (SD 2.09), with a median of 200 cells/μL (range 0 to 3,020) and an IQR of 110 to 300 cells/μL. The mean relative eosinophil level (percentage of white blood cells) was 2.38 (SD 2.35). Divided by blood eosinophil count, 28 patients (14%) had <100 cells/μL, 118 patients (59%) had between 100 and 299 cells/μL, and 54 (27%) had ≥300 cells/μL.

### 3.3 Risk factors

The cohort divided by subgroups of COPD risk factors is described in Table 2. Smoking, alone or with concurrent biomass exposure, was the most common risk factor (63%). Exposure to biomass smoke was also high (52.5%). The proportion of women in the biomass exposure group was more than three times higher than in the smoking group. Obesity was less frequent in the smoking exposure group compared to the other two. There were no significant clinical differences between the subgroups in Charlson Comorbidity Index, mMRC scale or GOLD classification.

**Table 2:** Description of the cohort based on COPD risk factors.

Characteristic	COPD risk factor *					
	Biomass		Biomass and smoking		Smoking	
	*n* = 62		*n* = 43		*n* = 83	
Age, years (mean, SD)	78.5	7.7	77.4	7.8	77.7	6.6
Gender, female (*n*, %)	49	79.0	21	48.8	18	21.7
Body mass index category (*n*, %)						
Normal weight	16	25.8	7	16.3	38	45.8
Overweight	21	33.9	20	46.5	25	30.1
Obesity	25	40.3	16	37.2	20	24.1
mMRC dyspnea score (*n*, %)						
1	0	0.0	2	4.7	1	1.2
2	48	77.4	28	65.1	61	73.5
3	14	22.6	13	30.2	21	25.3
Exacerbations in the previous year (*n*, %)						
One or more moderate	24	38.7	20	46.5	39	47.0
One or more severe	2	3.2	3	7.0	10	12.1
GOLD 2017 classification (*n*, %)						
B	51	82.3	35	81.4	60	72.3
C	0	0.0	2	4.7	1	1.2
D	11	17.7	6	14.0	22	26.5
GOLD 2023 classification (*n*, %)						
B	51	82.3	35	81.4	60	72.3
E	11	17.7	8	18.6	23	27.7
Eosinophil count, cells/μL (geometric mean, SD)	172.54	1.90	157.28	2.05	244.76	2.20
Relative eosinophil count (geometric mean, SD)	1.95	2.63	2.15	1.95	2.91	2.31
Exacerbations in the follow up year (*n*, %)						
One or more moderate	24	38.7	15	34.9	33	39.8
One or more severe	4	6.5	8	18.6	11	13.3

*Twelve patients with only occupational exposure were not included in the analysis. Patients with occupational exposure in addition to biomass, smoking or both, were included in the respective groups.

Although the highest mean blood eosinophil count was found in patients with a history of smoking, no trend was observed in the remaining groups that might suggest a relationship between risk factors and eosinophil levels. The subgroup with a history of smoking had a higher frequency of exacerbations in the year prior to the index date, but this difference was not observed in the follow-up year.

### 3.4 Clinical phenotype

Characteristics of the cohort according to clinical phenotype are summarized in Table 3. The most frequent clinical COPD phenotype was the non-exacerbator (78%). No differences were observed between phenotypes in airflow limitation, as 59% of patients were severe or very severe in all phenotypes. FEV_1_ and FVC values were also similar. Blood eosinophil count was higher in the frequent exacerbator phenotype. Most ACO patients were diagnosed due to a history of asthma (74%); only 7 patients were classified by reversibility ≥300 mL to beta 2 agonist on spirometry. Among the 27 patients with ACO, 5 also had an exacerbator phenotype.

**Table 3:** Description of the cohort according to COPD clinical phenotype.

Characteristic	Clinical COPD phenotype					
	Non-exacerbator		Frequent exacerbator		Asthma-COPD overlap	
	*n* = 156		*n* = 44		*n* = 27*	
Age, years (mean, SD)	77.9	7.5	78.4	7.2	73.9	8.3
Sex, female (*n*, %)	79	50.6	17	38.6	18	66.7
Body mass index categories (*n*, %)						
Normal weight	48	30.8	16	36.4	6	22.2
Overweight	52	33.3	17	38.6	9	33.3
Obesity	56	35.9	11	25.0	12	44.4
Charlson Comorbidity Index (median, IQR)	6	2.0	5	3.0	5	2.0
COPD risk factors (*n*, %)						
Smoking only	41	26.0	16	36.0	2	7.0
Biomass only	40	26.0	8	18.0	5	19.0
Occupational only	10	6.0	2	5.0	5	19.0
Biomass and smoking	24	15.4	4	9.1	9	33.3
Biomass and occupational	11	7.1	3	6.8	3	11.1
Occupational and smoking	19	12.2	7	15.9	1	3.7
Biomass and smoking and occupational	11	7.1	4	9.1	2	7.4
mMRC dyspnea score (*n*, %)						
1	0	0.0	3	6.8	1	3.7
2	120	76.9	25	56.8	22	81.5
3	36	23.1	16	36.4	4	14.8
FEV_1_, L (mean, SD)	1.01	0.38	1.03	0.39	1.11	0.39
FEV_1_, % predicted (mean, SD)	53.7	21.6	49.7	17.2	52.2	20.9
FVC, L (mean, SD)	1.88	0.61	1.98	0.65	1.96	0.67
FVC, % predicted (mean, SD)	73.2	23.0	74.2	20.3	71.1	26.7
Eosinophil count, cells/μL (geometric mean, SD)	193.4	2.12	212.2	2.00	173.8	2.07
Relative eosinophil count (geometric mean, SD)	2.31	2.44	2.67	2.03	2.37	2.08
Maintenance inhaler therapy at baseline (*n*, %)						
LAMA or LABA in monotherapy	10	6.4	3	6.8	1	3.7
LABA and LAMA combination	15	9.6	5	11.4	2	7.4
ICS and long-acting bronchodilator combination	49	31.4	12	27.3	11	40.7
Triple therapy (ICS, LABA and LAMA)	64	41.0	23	52.3	9	33.3
Other ^†^	18	11.5	1	2.3	4	14.8
Exacerbations in the follow up year (*n*, %)						
One or more moderate	53	34.0	24	54.6	11	40.7
One or more severe	16	10.3	9	20.5	4	14.8

LAMA, long-acting muscarinic antagonist; LABA, long-acting β2-agonist; ICS, inhaled corticosteroid.

*Asthma-COPD overlap is not exclusive; 22 and 5 out of 27 patients had non-exacerbator and frequent exacerbator phenotypes, respectively; they were counted in those groups as well.

^†^Other combinations included short-acting bronchodilators with or without an ICS.

### 3.5 Treatment patterns

At baseline, 44% of the patients were receiving triple therapy (inhaled corticosteroid [ICS] plus long-acting β2-agonist [LABA] and long-acting muscarinic antagonist [LAMA]) followed by 30% with double therapy with ICS + LABA (delivered in single or multiple inhalers), 10% with double therapy with LABA+LAMA (delivered in single or multiple inhalers) and 7% with monotherapy with long-acting bronchodilators. During the follow-up year, the use of long-acting bronchodilators increased, either in monotherapy or in LABA+LAMA combination, while the use of ICS + LABA (from 30.5 to 25.5%) and triple therapy (from 43.5 to 38.5%) decreased (Table 4).

**Table 4:** COPD maintenance therapy during the observation year.

Treatment	Baseline		6 months		12 months	
	*n*	%	*n*	%	*n*	%
LAMA or LABA in monotherapy	13	6.5	17	8.5	21	10.5
LABA and LAMA combination	20	10	28	14	38	19
ICS and long-acting bronchodilator combination	61	30.5	64	32	51	25.5
Triple therapy (ICS, LABA and LAMA)	87	43.5	79	39.5	77	38.5
Other*	19	9.5	12	6	13	6.5

LAMA, long-acting muscarinic antagonist; LABA, long-acting β2-agonist; ICS, inhaled corticosteroid.

*Other combinations included short-acting bronchodilators with or without an ICS.

In the cohort, 177 (88.5%) patients were vaccinated against Influenza and 152 (76%) against pneumococcal infections. Long-term home oxygen requirement was remarkably high (190 patients, 95%), probably attributed to the cohort’s disease severity and location in the high-altitude city of Bogotá, Colombia (2,625 meters above sea level). In contrast, only 2.5% were referred to a pulmonary rehabilitation program.

### 3.6 Clinical outcomes

A total of 142 exacerbations were recorded in the year before the index date, of which 124 were moderate (87.3%) and 18 severe (12.7%). Eighty-eight patients (44%) had one or more moderate exacerbations, and 18 patients (8%) had at least one severe episode. During the follow-up year, exacerbations were even more frequent, with 152 events, 122 (80.3%) moderate and 30 (19.7%) severe. Seventy-seven patients (38.5%) had one or more moderate exacerbations, and 25 (12.5%) had one or more severe events. The annual rate of moderate and severe exacerbations was 0.61 (SD 0.87) and 0.15 (SD 0.43), respectively. One death was observed 11 months after onset, an 81-year-old woman with prior biomass exposure, GOLD B classification, non-exacerbator phenotype, and arterial hypertension as comorbidity.

The moderate exacerbation rate in the year before the index date was 0.56 (SD 0.75) in the ACO phenotype and 0.63 (SD 0.82) in the non-ACO phenotypes. During the follow-up year, rates did not change, with 0.56 (SD 0.75) in the ACO phenotype and 0.62 (SD 0.89) in the non-ACO phenotypes. Severe exacerbation rates increased in both ACO and non-ACO patients from 0.1 (SD 0.34) and 0.04 (SD 0.19) at baseline to 0.15 (SD 0.45) and 0.14 (SD 0.36) in the following year, respectively.

Patients in the non-exacerbator phenotype had a similar risk of moderate exacerbations in the years before and after the index date (32.7 and 34% respectively). In addition, 10% of patients in this group had severe exacerbations during the follow-up year. In contrast, in the patients with the frequent exacerbator phenotype, the probability of having one or more exacerbations was higher in the year before the index date compared to the follow-up year (84.1% vs. 54.5%, respectively, for moderate, and 36.4% vs. 20.4% for severe). The annual exacerbation rate during the follow-up year showed a trend toward a higher frequency of exacerbations in patients with higher eosinophil counts and the frequent exacerbator phenotype, although the data showed significant variability. Table 5 describes the frequency of exacerbations according to clinical phenotypes and eosinophil level categories.

**Table 5:** Frequency of exacerbations by clinical phenotypes and eosinophil level categories.

Baseline eosinophil level	Clinical phenotype		
	Non-exacerbator	Frequent exacerbator	Total
<300	117	29	146
	61 / 17	19 / 6	80 / 23
	0.67 (0.94)	0.86 (0.79)	0.71 (0.91)
≥300	39	15	54
	24 / 4	18 / 3	42 / 7
	0.72 (0.83)	1.40 (0.99)	0.91 (0.92)
Total	156	44	200
	85 / 21	37 / 9	122 / 30
	0.68 (0.91)	1.05 (0.89)	0.76 (0.91)

Top value: N, Middle value: number of moderate / severe exacerbations, Bottom value: mean (standard deviation) of total exacerbations.

### 3.7 Association between eosinophil levels and exacerbations

The results of the *post hoc* Poisson regression are presented in Table 6. In summary, while controlling for sex and smoking status, having both the frequent exacerbator phenotype and blood eosinophils ≥300 cells/μL at baseline significantly increased the rate of moderate to severe exacerbations during the observation year (Incidence Rate Ratio [IRR] 2.17, 95% Confidence Interval [CI] 1.31 to 3.59, *p* = 0.003) compared to patients with eosinophils <300 and the non-exacerbator phenotype. Despite the positive interaction, an eosinophil count ≥300 cells/μL was not independently associated with exacerbation rate (IRR 1.1, 95% CI 0.71 to 1.71, *p* = 0.657).

**Table 6:** Results of the Poisson regression model.

Variable	IRR	SE	*p*-value	LB 95% CI	UB 95% CI
EOS ≥ 300 cells/μL (versus EOS < 300 cells/μL)	1.10	0.247	0.657	0.71	1.71
Frequent exacerbator phenotype (versus non-exacerbator)	1.31	0.301	0.244	0.83	2.05
Interaction of EOS ≥ 300 cells/μL and frequent exacerbator phenotype (versus EOS < 300 cells/μL and non-exacerbator phenotype)	2.17	0.558	0.003	1.31	3.59
Smoking status (current or past smoker versus never smoker)	1.06	0.205	0.749	0.73	1.55
Sex (male versus female)	0.86	0.158	0.418	0.60	1.23
Constant (baseline incidence rate)	0.685	0.106	0.015	0.506	0.928
**Poisson model evaluation**					
Non-events: 103 (51%)					
Mean: 0.76					
Variance: 0.956					
Dispersion of complete model: Pearson = 1.13					

Each table contains the relative change in the incidence rate of moderate to severe exacerbations of the clinical characteristic, adjusted by the other variables in the model.

IRR, Incidence Rate Ratio; SE, standard error; LB, lower boundary; UB, upper boundary; CI, confidence interval; EOS, baseline blood eosinophil count.

## 4 Discussion

Several cohorts published in the last decade have allowed us to characterize phenotypes and treatable traits in COPD patients (3, 21, 22). This study provides a characterization and analysis of eosinophil levels and exacerbations in COPD patients with moderate to severe COPD from comprehensive programs living at high altitude in Colombia. Regarding socio demographic data of this cohort, it is noteworthy that 88% of patients were older than 70 years, with a mean age of 77.9 years, higher than other COPD cohorts (mean age was 63 years in ECLIPSE and 65.2 years in SPIROMICS) (22, 23). Remarkably, more than half of the patients included had exposure to biomass as a risk factor for the development of COPD, a risk factor not reported in the reference studies. In both the ECLIPSE and SPIROMICS cohorts, the risk factor for COPD among included patients was specified as individuals who were current or former smokers with a cumulative smoking history of ≥10 pack-years; there are no reported data regarding biomass as a risk factor for COPD (22, 23). This exposure has a significant relevance in developing countries, where biomass and coal serve as vital sources of fuel within the household (24). Particularly, in Colombia wood smoke-induced COPD is a common condition significantly different from COPD caused by tobacco smoke, as it has more inflammatory airway involvement related to chronic bronchitis, bronchial hyperresponsiveness, and significantly less emphysema (8, 25, 26).

Despite neutrophilic COPD being the prevailing inflammatory phenotype, a significant portion of COPD patients (ranging from 10 to 40%) exhibit heightened eosinophilic inflammation in their sputum or blood samples. This increase in eosinophilic inflammation is accompanied by elevated Th-2 transcriptomic signatures, regardless of whether the patients have a history of asthma (27, 28). Identifying eosinophil levels is relevant as a predictor of acute exacerbations and has emerged as a biomarker that can help guide decisions about pharmacological treatment (17, 29). This study included eosinophil levels, risk factors for developing COPD and clinical phenotypes. The geometric mean blood eosinophil count was 197 cells/μL, which is comparable to the results reported in the IMPACT trial (170 cells/μL [IQR 90–270]) (12) and the ECLIPSE cohort (150 cells/μL) (22).

The value of blood eosinophils may be limited in areas with endemic helminth parasitic infections, especially in developing countries. However, some studies in these settings have shown that eosinophil counts are not affected by the prevalence of these infections and could therefore be helpful as a biomarker in COPD (30).

In terms of phenotypic stratification, there were differences in smoking history among the phenotypes evaluated in the cohort. Smoking as a risk factor was higher in the frequent exacerbator phenotype compared to the non-exacerbator phenotype (36% vs. 26%). The blood eosinophil count was higher in the frequent exacerbator phenotype, validating its usefulness as a predictor of exacerbations, but was lower in the ACO phenotype, even though it was expected that these patients would demonstrate features of Th-2 inflammation (31). In our cohort, patients with ACO phenotype were more likely to be female, younger, and exposed to biomass. In the PUMA study that included Colombian COPD patients, those with ACO were similar in age, gender, biomass smoke exposure and pack-years of smoking compared with other groups of COPD (32). As expected, all patients with the non-exacerbator phenotype were classified as GOLD B, and all patients with the frequent exacerbator phenotype were classified as GOLD E. Although previous reports have found that ACO patients had more symptoms and exacerbations (33), in our cohort only 18% of patients with ACO were classified as GOLD E.

Overall, severe exacerbations during the follow-up year were more frequent than in the previous year. This increase was mainly caused by occurrence of new events in the non-exacerbator phenotype subgroup, which were 78% of the total study population. In contrast, patients with the frequent exacerbator phenotype shown a crude decrease in exacerbations in the follow-up year. A possible explanation for this is the specific therapy received for each phenotype in specialized centers. Concerning treatment, due to their attendance to specialized centers with integrated COPD programs, patients from this cohort had considerably different patterns of therapy compared to other studies in Colombia (33, 34), which have shown a lower use of ICS + LAMA+LABA combinations and a higher use of therapies exclusively based on short-acting medication. Inhalers containing combinations with ICS have been reported as the most widely used medications in Latin American countries, independent of the GOLD classification (33).

Despite the relatively low cohort size, the findings of the *post hoc* regression analysis are consistent with those published in the literature, which report having blood eosinophils ≥300 cells/uL and history of frequent exacerbations as predictors of future events (19, 35, 36). The clinical repercussions and biological plausibility of the interaction found need to be evaluated in future studies.

Our study has some limitations. First, the clinical data collected were based on medical records, which may introduce misclassification or recall biases. Specifically, COPD severity or comorbidities might be underestimated in our records. Additionally, data regarding clinically important factors such as alpha-1 antitrypsin and fractional exhaled nitric oxide (FENO) was not available in the clinical records. As of the study’s completion date in 2019, guidelines did not recommend systematic screening for alpha-1 antitrypsin deficiency due to its low prevalence on the global population (37). On the other hand, FENO was not available in the country during the time the cohort was diagnosed and treated. Second, as is common in retrospective studies, the exclusion of patients with incomplete follow-up could have introduced a selection bias in the outcomes when missing data did not occur at random. Third, due to the fragmentation of the Colombian healthcare system and the lack of national electronic health records, the study may have missed some events that happened outside the study centers and were not reported by the patients during their medical visits, leading to a potential underestimation of exacerbations and deaths. Fourth, our study population was limited to patients with moderate to severe COPD attending a comprehensive disease management program between 2015 and 2019 in the high-altitude city of Bogotá, which is not representative of the whole Colombian COPD population. Finally, the low number of selected patients compared to other cohorts limited the study’s potential to find statistically significant associations.

In conclusion, in a cohort of patients with moderate to severe COPD, the combination of frequent exacerbator phenotype and a blood eosinophil count greater than 300 cells/μL at baseline were associated with a higher risk of exacerbations in the following year. Mean eosinophil count was comparable to previously published cohorts and was higher in patients with a history of smoking and frequent exacerbator phenotype. In this cohort of COPD patients, biomass exposure was identified as a predominant risk factor.

## Data availability statement

The datasets presented in this study can be found in online repositories. The names of the repository/repositories and accession number(s) can be found at: https://www.gsk-studyregister.com/en/ 207254.

## Ethics statement

The studies involving humans were approved by Comité de Investigaciones y Ética Institucional, Facultad de Medicina, Pontificia Universidad Javeriana, Hospital Universitario San Ignacio. The studies were conducted in accordance with the local legislation and institutional requirements. The ethics committee/institutional review board waived the requirement of written informed consent for participation from the participants or the participants’ legal guardians/next of kin because by Colombian regulation, retrospective studies that do not pose a risk to its participants can receive a waiver from obtaining informed consent from the Ethics Committee that approved the study.

## Author contributions

OG: Conceptualization, Investigation, Writing – original draft, Writing – review & editing. AC-A: Conceptualization, Writing – review & editing, Investigation. MR: Formal analysis, Methodology, Writing – review & editing, Conceptualization. JG: Conceptualization, Investigation, Writing – review & editing. PR: Conceptualization, Investigation, Writing – review & editing. VA: Conceptualization, Formal analysis, Methodology, Writing – review & editing. AG-R: Supervision, Writing – original draft, Writing – review & editing, Project administration, Methodology, Funding acquisition, Conceptualization. CC-P: Conceptualization, Investigation, Writing – review & editing.
